# Randomized Controlled Trial of Paliperidone Extended Release Versus Risperidone for the Treatment of Methamphetamine-Associated Psychosis in Chinese Patients

**DOI:** 10.3389/fpsyt.2020.00237

**Published:** 2020-04-01

**Authors:** Gang Wang, Fan Ding, Marek Cezary Chawarski, Wei Hao, Xuebing Liu, Qijian Deng, Xuan Ouyang

**Affiliations:** ^1^Affiliated Wuhan Mental Health Center, Tongji Medical College of Huazhong University of Science & Technology, Wuhan, China; ^2^Wuhan Wudong Hospital, The Second Mental Hospital of Wuhan, Wuhan, China; ^3^Department of Psychiatry, Yale School of Medicine, New Haven, CT, United States; ^4^Key Laboratory of Psychiatry and Mental Health of Hunan Province, China National Clinical Research Center for Mental Health Disorders, Mental Health Institute of the Second Xiangya Hospital, National Technology Institute of Psychiatry, Central South University, Changsha, China

**Keywords:** methamphetamine, psychosis, paliperidone extended-release, risperidone, efficacy, safety

## Abstract

**Background:**

The efficacy or tolerability of paliperidone extended release (ER) in the treatment of methamphetamine (METH)-associated psychosis (MAP) is unknown. This study was designed to assess the tolerability and efficacy of paliperidone ER and risperidone for the treatment of MAP in China.

**Methods:**

This 25-day randomized clinical trial involved 120 patients with acute MAP symptoms who were randomized to receive either paliperidone ER or risperidone from baseline to day 25 of an inpatient hospital stay. The primary outcome was changes in the severity of psychosis, which were assessed using the Positive and Negative Syndrome Scale (PANSS) total score changes from baseline to endpoint.

**Results:**

Overall, 84% of the patients completed the entire study protocol. The PANSS total score, the Clinical Global Impressions-Severity of Illness scale (CGI-S) score, and a METH craving score assessed by a visual analog scale (VAS) showed statistically significant improvements from baseline for the patients in both groups (*p* < 0.01). The Simpson-Angus Scale (SAS) and the Barnes Akathisia Rating Scale (BARS) scores increased from baseline during treatment in both groups (*p* < 0.01); there were statistically significant differences between the treatment groups in the SAS scores (*p* < 0.01). Measures of hypermyotonia, salivation, and dizziness were significantly higher in the risperidone-treated patients than in the paliperidone ER-treated patients (all *p* < 0.05).

**Conclusion:**

Paliperidone ER and risperidone had similar efficacy and were generally tolerable in the treatment of MAP; however, paliperidone ER had a more favorable adverse event profile than risperidone, particularly regarding extrapyramidal and prolactin-increasing effects.

**Clinical Trial Registration:**

ClinicalTrials.gov, identifier NCT01822730. Full date of first registration:03/28/2013.

## Introduction

The use of methamphetamine (METH) and amphetamine-type stimulants (ATS) is a significant global problem, with an estimated 34 million people using ATS in 2016 (1). In China, the number of registered ATS users has increased dramatically in recent years, and METH use has been reported by approximately 78% of registered ATS users (2). METH-associated psychosis (MAP) is common and includes hallucinations and delusions, which result in disability, substantial loss of life and increased rates of crime, violence, and emergency room/trauma center visits (3–5). Most individuals with MAP have transient psychosis but remain at risk for persistent psychosis. METH could resulted in gray matter reductions in the anterior cingulate have been reported as markers of genetic liability to psychosis, while reductions in the superior temporal gyrus and cerebellum may be interpreted as markers of a first onset of the MAP (6). In addition, stigmatization of psychosis is widespread, and its genetic explanation may potentially increase the stigma. In particular, considering schizophrenia as a genetic disorder influenced participants’ perception of other people’s beliefs about dangerousness and unpredictability and people’s desire for social distance. Some studies showed that there were high levels of perceived stigmatization in medical students and medical doctors and at least half of the analyzed subjects perceived stigmatizing social attitudes against psychotic individuals (7). Therefore, it is important to prevent and treat MAP as well as primary psychotic disorders, such as schizophrenia (8).

Antipsychotics, both typical and atypical, are often used for the treatment of MAP. Several clinical trials have reported that antipsychotics, such as aripiprazole, risperidone, haloperidol, and quetiapine, are effective for patients with MAP, but therapeutic effects and adverse events might differ among those agents (9–11). We also found that risperidone might be an effective antipsychotic treatment for MAP with adverse events comparable to those of treatment with aripiprazole; however, obvious adverse events, such as dystonia and sialorrhea, still existed in the risperidone group (12).

Paliperidone, also referred to as 9-hydroxyrisperidone, is the major active metabolite of risperidone (13) and is used in the treatment of schizophrenia and related disorders (14, 15). Despite their parent/metabolite relationship, these two agents have distinct pharmacokinetic and other biochemical characteristics that may result in differences in therapeutic efficacy or safety. Specifically, paliperidone ER with an osmotic-controlled release oral delivery system minimizes drug plasma fluctuations and may have a reduced risk of unexpected overdosage relative to oral risperidone (16). Additionally, hepatic metabolism has a limited role in the excretion of paliperidone ER compared with risperidone, which may reduce the likelihood of underdosage due to CYP2D6 genetic variability (17). Compared with risperidone, paliperidone ER has a faster off-rate for dissociation from human-cloned D2 receptors in tissue culture cells (18). Because of its looser binding to D2 receptors and its weaker affinity for H_1_, α1-, and α2-adrenergic receptors in the brains of animals, paliperidone ER should be associated with a reduced risk of extrapyramidal symptoms (EPS) and sedative effects and lower weight gain compared with its parent drug (19, 20). In addition, paliperidone ER may have improved medication adherence due to the reduced frequency of administration (once per day) relative to oral risperidone (twice per day). Overall, these characteristics suggest a potentially improved efficacy and better adverse effect profile with paliperidone ER compared with risperidone.

Recent studies (21) demonstrated that paliperidone ER is efficacious, safe, and well accepted when compared with other pooled second-generation antipsychotic drugs for the treatment of Chinese patients with schizophrenia. A double-blind, placebo-controlled trial of risperidone and paliperidone ER with healthy volunteers suggested that compared to risperidone, paliperidone ER may have a better safety profile in terms of negative subjective experiences and cognitive function among normal volunteers and patients with schizophrenia (22, 23).

To date, however, no studies have been conducted to investigate potential differences in efficacy and tolerability between paliperidone ER and oral risperidone in the treatment of MAP. We hypothesized that paliperidone ER would have a better efficacy and safety profile than risperidone in the treatment of MAP. Therefore, the main objective of this study was to assess psychotic symptoms in individuals with MAP in response to multiple doses of paliperidone ER and risperidone in a randomized controlled trial. Adverse events caused by these agents were also evaluated.

## Methods

### Study Overview

The study was conducted in the inpatient ward of the Wuhan Mental Health Center in Wuhan, Hubei Province, China. The Wuhan Mental Health Center is the largest mental hospital in Hubei Province, with 530 hospital beds and a total of 2,000 admissions per year. The inpatient ward that is dedicated to the voluntary treatment of MAP treats approximately 65 patients at any given time, with a total of 600 to 800 admissions per year. The current study enrolled patients and provided study-related patient care and evaluation between February 2013 and March 2014. The trial was sponsored and authorized by the Department of Psychiatry and Mental Health Institute of the Second Xiangya Hospital, Central South University. This study was performed in accordance with the provisions of the Declaration of Helsinki, and the study protocols and procedures were approved by the Second Xiangya Hospital ethics committees. The trial was registered at ClinicalTrials.gov (See Trial Registration in details) and complied with SPIRIT guidelines. All patients provided written informed consent.

### Study Design

This was a randomized, 25-day, assessor-blinded, parallel-group trial of MAP inpatients. Assuming that the mean Positive and Negative Syndrome Scale (PANSS) total score would be decreased to 30 in the paliperidone ER group and to 40 in the risperidone group from baseline to day 25, we calculated that a sample size of 100 patients per treatment group would provide 95% power with 10 repeated measurements, with a correlation of 0.5 between observations in the same subject and an alpha level of 0.05. The sample size was inflated by 20% to account for loss to follow-up. The total sample size was 120, with 60 in each group (9, 24, 25).

### Study Participants

Study eligibility criteria included men and women aged 18 to 60 years with a DSM-IV diagnosis of MAP. A diagnosis of substance-induced psychotic disorder was made when the following symptoms were present: 1) prominent hallucinations or delusions; a score ≥ 4 on at least one PANSS (26) psychosis item (delusions, conceptual disorganization, hallucinatory behavior, grandiosity, or suspiciousness/persecution) and a score ≥4 (moderately ill) on the severity item of the CGI-S at the point of maximum illness severity to date; 2) hallucinations or delusions that developed during or soon after intoxication with or withdrawal from a substance or medication known to cause psychotic symptoms; duration of psychotic symptoms for more than 2 weeks; 3) psychotic symptoms that are not part of a psychotic disorder (such as schizophrenia, schizophreniform disorder, or schizoaffective disorder) that are not substance induced (i.e., if psychotic symptom onset was prior to substance or medication use or persists longer than 1 month after substance intoxication or withdrawal, then another psychotic disorder is likely); 4) psychotic symptoms that do not only occur during a delirium; and 5) use of METH at least once per week during the three months prior to enrollment and a positive urine screen for METH. Participants were also required to not be dependent on substances other than nicotine and not experiencing acute METH intoxication effects that could interfere with the informed consent process or compliance with study procedures. The presence of serious medical illnesses, such as physical disease, mental illnesses that require treatment with antipsychotics, or with suicidal ideation and/or a positive history of previous suicide attempts were the exclusion criterion. Female participants with child-bearing potential needed to be using a medically acceptable form of contraception.

### Study Medication

During the first 7 days of treatment, patients assigned to treatment with paliperidone ER initially received 3 mg per day in the morning; the daily dose could be increased to up to 9 mg per day at the discretion of the attending physician based on the severity of psychotic symptoms or the response to the initial dose of medication. For the remaining 18 treatment days, the highest dose received during the first 7 days was maintained as the daily treatment dose. In the paliperidone ER group, 9 participants reached doses of 3 mg, 49 reached 6 mg, and 2 reached 9 mg per day.

A similar protocol was applied for risperidone patients: they initially received a daily dose of between 1 and 2 mg in a twice-a-day dosing regimen (morning and evening). Based on the patient’s clinical response and tolerance, the risperidone could be increased to up to 3 mg twice a day during the first 7 days of treatment by the attending physician. For the remaining 18 treatment days, the highest risperidone dose received during the first 7 days was maintained as the daily treatment dose. In the risperidone group, 17 participants reached doses of 3 mg, 27 reached 4 mg, 8 reached 5 mg, and 8 reached 6 mg per day.

In addition to receiving medication treatment, the inpatients also received cognitive-behavioral therapy and music therapy to aid the recovery process and help address their cognition, behavior and emotion regulation. Both types of therapies were provided in 30- to 45-minute group sessions once per week and were conducted by specialty-trained clinicians. The patients also had access to a gymnasium and unscheduled leisure time activities, such as playing chess and cards or reading books in designated areas of the ward. The patients were housed in pairs in a shared room with a TV and bathroom, and their meals were ordered from and provided by the hospital cafeteria. The inpatients were not allowed to leave the ward, but their family members could visit them in the ward once per week for a half day.

### Randomization and Blinding Procedures

A study statistician with no involvement in the clinical or research activities during the study generated a 1:1 random allocation sequence using a fixed-block size of 8 and put the allocation sequence in sequentially numbered, opaque, sealed, and stapled envelopes. We separated the staff who performed the assessments (PANSS, CGI-S, METH craving, EPS) from the staff who delivered the intervention. The assessors had been trained before making the assessments and were not informed of the medication group assignment. After the training sessions, 85% of the PANSS items reached an acceptable level of reliability (kappa(w) > 0.50). The treatment intervention staff and psychologists did not perform any of the assessments. While the study participants were not informed which medication they received, the medications were not blinded or overencapsulated, and the staff who delivered the intervention knew the medication group assignment of each patient.

### Procedures

Screening was performed by clinical staff when the patients were on the ward. The screening included complete histories and physical information, blood counts, metabolic panels, liver function tests, and rapid qualitative urine METH tests using immunochromatographic METH metabolite detection. Then, at the baseline clinic visit, randomization and drug dispensing occurred. We planned to follow the patients for 25 days during their inpatient stay with frequent assessments of psychotic symptoms and potential adverse effects (on days 3, 5, 7, 10, 13, 16, 19, 22, and 25 during the hospital stay). At each time point over the 25 days, psychotic symptoms were assessed, and a METH craving assessment and a physical exam were conducted. Safety labs were performed at baseline and at the 25-day time point.

### Outcomes

#### Primary Outcome

The severity of psychosis were assessed using the PANSS total score from baseline to endpoint.

#### Secondary Outcomes

The overall severity of the illness were evaluated with the CGI-S. METH craving during the study were assessed by a weekly self-reported visual analog scale (VAS) score of the need for METH (scale 0–10; 0, not at all; 10, very much so).

#### Safety Assessments

The severity of EPS was assessed by the Barnes Akathisia Rating Scale (BARS) (27) and the Simpson-Angus Scale (SAS) (28). At each follow-up, vital signs and body weight were also measured. Changes in the patients’ blood chemistry, and electrocardiogram (ECG), were also evaluated for the study completers.

### Statistical Analyses

Baseline differences between the two randomized groups were evaluated using independent *t*-tests for continuous variables and chi-square tests for categorical variables. Differences in retention were evaluated using a Kaplan-Meier survival analysis. For each study participant, the means of three consecutive assessments during treatment were computed approximately weekly during inpatient treatment and used as the repeated assessment measures. Primary and secondary outcomes and safety were used in the linear mixed model and repeated-measures analyses to evaluate the statistical significance of group, time, and group by time interaction effects on measures of psychotic symptoms, METH craving, EPS, and weight (29). Differences in the incidence of adverse events between the two treatment groups were evaluated using chi-square tests. Changes in the patients’ blood chemistry and ECG from baseline to day 25 were evaluated using independent *t*-tests.

## Results

### Screening, Randomization, and Participant Characteristics

**Figure 1:**
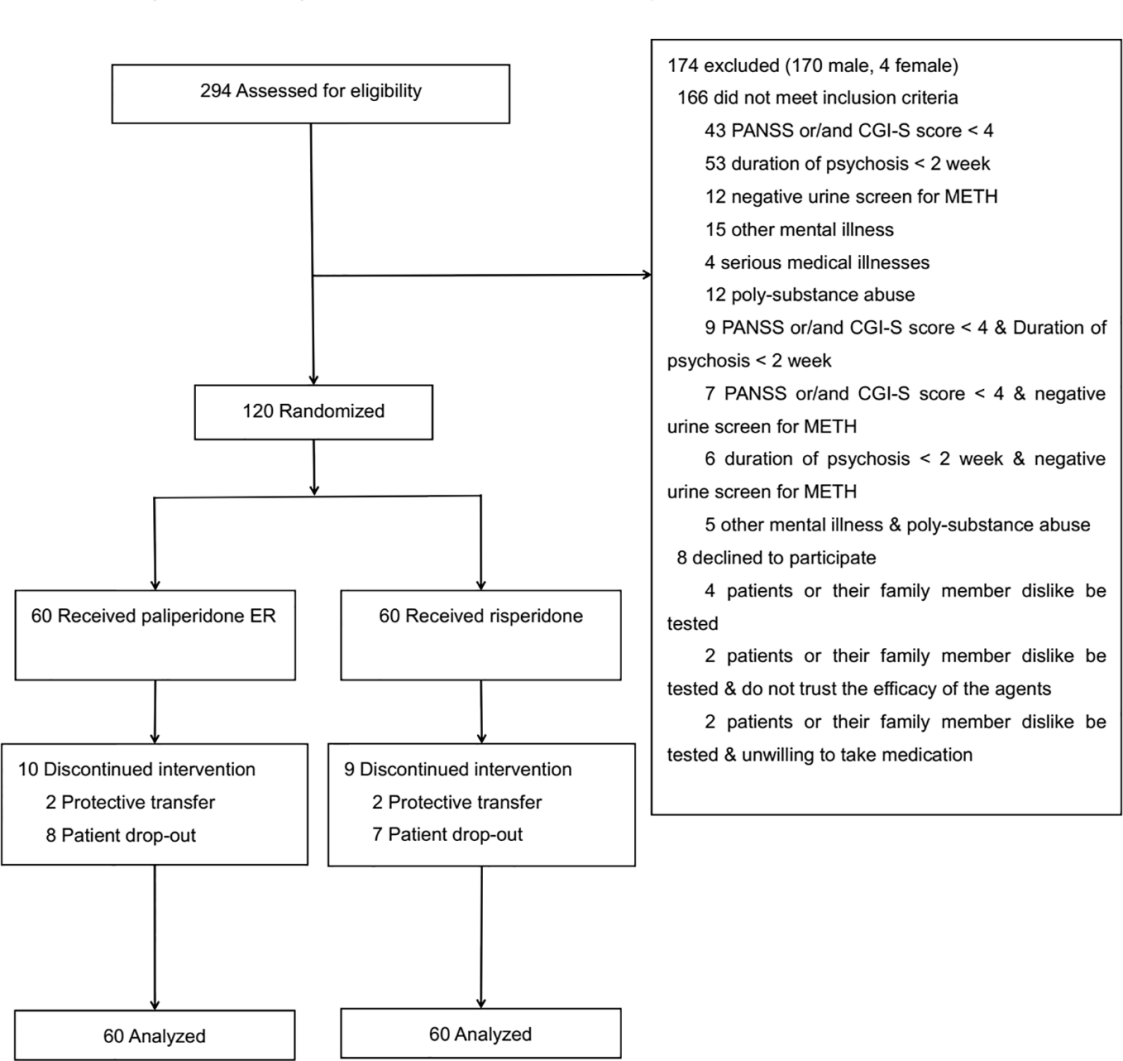
Screening, randomization, and follow-up of study participants.

During the active study period (from February 2013 to March 2014), a total of 637 patients were admitted to the ward. Of these, 294 were screened for initial eligibility, and 166 were found ineligible. Of the 128 eligible individuals, 120 agreed to participate and were randomized into the trial. Those who were eligible but did not participate in the trial were similar in age, race/ethnicity, and METH use to those who were randomized. See **Figure 1** for the CONSORT diagram.

The majority of the study participants were men (109/120, 90.8%), the mean (SD) age of the subjects was 31.2 (7.5) years, and 69/120 (57.5%) reported using METH 3 to 6 days per week prior to hospital admission. There were no significant differences in baseline characteristics or baseline clinical evaluation data between the two treatment groups (*p* > 0.05 for all comparisons) except for the frequency of METH use in the past 4 weeks and the METH craving score (*p* ≤ 0.01). The baseline characteristics of the study participants are presented in **Table 1**.

**Table 1:** Demographics and baseline clinical characteristics of the enrolled sample (N = 120).

Demographics	Paliperidone ER	Risperidone	*p*
	n = 60	n = 60	
Age, mean (SD), y	31.1 (7.9)	31.2 (7.2)	0.3
Gender, n (%)			
Male	57 (95.0)	52 (86.7)	0.1
Female	3 (5.0)	8 (13.3)	
Race/ethnicity			
Chinese	60 (100)	60 (100)	1
Marital status (n, %)			
Married	33 (55.0)	35 (58.3)	0.8
Never married	23 (38.3)	20 (33.3)	
Divorced	4 (6.7)	5 (8.3)	
Education (n, %)			
Tertiary (16 or more years)	10 (16.7)	3 (5.0)	0.1
Secondary (12 years)	44 (73.3)	50 (83.3)	
Primary (6 years)	6 (10.0)	7 (11.7)	
Income, mean (SD) (in Renminbi per month)	10633.3 (16414.0)	10508.3 (12448.4)	1.0
Employment status (n, %)			
Not employed	28 (46.7)	31 (51.7)	0.8
Part time	3 (5.0)	2 (3.3)	
Full time	29 (48.3)	27 (45.0)	
**METH use**			
Onset age, mean (SD)	26.8 (7.8)	27.7 (7.2)	0.5
Duration used, mean (SD), years	4.3 (2.4)	3.9 (2.3)	0.3
Frequency of METH use in past 4 weeks, n (%)			
≤2 d/wk	14 (23.3)	9 (15.0)	0.01
3–6 d/wk	39 (65.0)	30 (50.0)	
7 d/wk	7 (11.7)	21 (35.0)	
Route of METH administration, n (%)			
Smoked	60 (100)	60 (100)	1
Nicotine dependence, n (%)	58 (96.7)	57 (95.0)	0.6
Alcohol abuse, n (%)	8 (13.3)	10 (16.7)	0.6
**Baseline clinical characteristics**			
PANSS total score (SD)	73.0 (12.3)	74.4 (12.8)	0.5
PANSS positive score (SD)	22.5 (4.4)	23.8 (4.9)	0.1
PANSS negative score (SD)	14.5 (5.6)	14.5 (6.0)	1.0
PANSS general psychopathology score (SD)	35.8 (6.4)	36.0 (6.5)	0.9
CGI-S	5.4 (0.8)	5.5 (0.8)	0.6
VAS	7.5(1.1)	6.5(1.7)	< 0.01
BARS	0.6(0.7)	0.5(0.8)	0.4
SAS	0.1(0.4)	0.1(0.5)	0.7
Weight, kg	66.0(12.6)	64.5(9.6)	0.5

METH, methamphetamine; SD, standard deviation; PANSS, Positive And Negative Syndrome Scale; CGI-S, Clinical Global Impression—Severity; BARS, Barnes Akathisia Rating Scale; SAS, The Simpson-Angus Scale; VAS, visual analog scale.

### Treatment Retention and Medication Adherence

**Figure 2:**
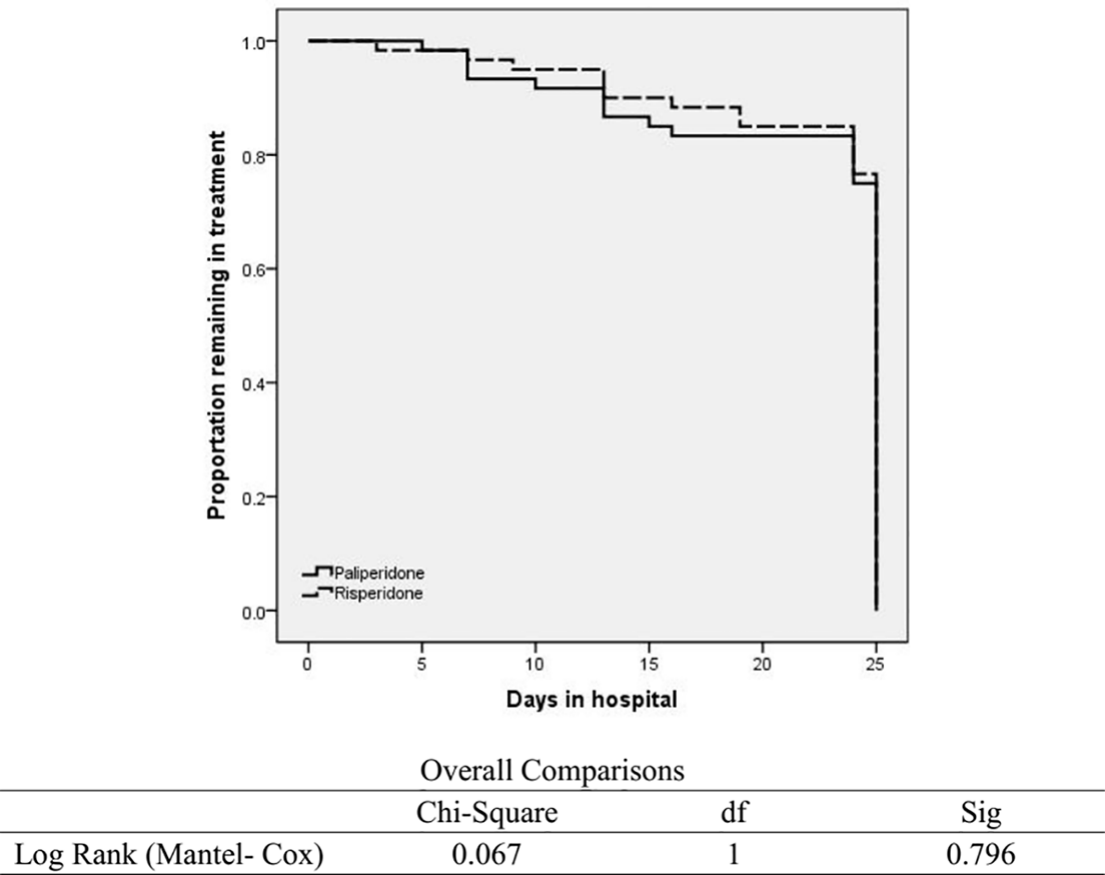
Kaplan-Meier survival curves with proportion of patients remaining in treatment in the two study group.

Overall, 101/120 (84%) patients completed the entire study protocol, and there were no statistically significant between-group differences in trial completion (22.5 days [95% CI, 21.1–23.9] in the paliperidone ER group vs. 23.0 days [95% CI, 21.8–23.7] in the risperidone group, *p* = 0.8). The data are also presented in **Figure 2**.

Ten patients in the paliperidone ER group and 9 patients in the risperidone group discontinued the study. Patient self-termination (dropout) from the study was the primary reason for discontinuation in both groups (8/10 or 80% of the paliperidone ER group vs. 7/9 or 78% of the risperidone group). Patients who did not respond to treatment and showed no improvements in their psychotic symptoms during the first two weeks of the treatment were protectively transferred to a standard clinical protocol: their study medications were discontinued, and they remained on the ward to be treated with clozapine (1 patient in the paliperidone ER group and 2 patients in the risperidone group). One patient in the paliperidone ER group was discontinued due to edema of the face, arms, and legs that developed during the first week of the study treatment. This patient was also protectively transferred to a standard clinical protocol: the study medications were discontinued, and the patient remained on the ward to be treated with olanzapine.

The participants took the medications on a schedule while face-to-face with a clinician and a nurse, who checked their mouths after 5 minutes to confirm that the medications had been swallowed. Therefore, the participants took all medications on time in our study.

### Efficacy

The PANSS total score; PANSS positive, negative, and general psychopathology scale scores; CGI-S score; and METH craving score showed statistically significant improvements from baseline over time for patients in both groups (*p* < 0.01), and the differences between the treatment groups were not statistically significant (*p* > 0.05 for the medication-by-time interaction effect; see **Table 2** for detailed results).

**Table 2:** Study outcomes in the two study groups at baseline and during treatment (N=120).

Outcome measure Mean (SD)	Baseline	Time 1 Days 1–7	Time 2 Days 10–16	Time 3 Days 19–25	Significance *p value*		
					Medication effect	Time effect	Medication×Time interaction
**PANSS total score**							
Paliperidone (n = 60)	73.0 (12.3)	57.4 (11.1)	44.3 (10.0)	36.9 (8.6)	<0.05	<0.01	0.06
Risperidone (n = 60)	74.4 (12.8)	61.6 (10.4)	46.4 (8.3)	39.4 (6.4)			
**PANSS positive score**							
Paliperidone (n = 60)	22.5 (4.4)	15.8 (4.5)	10.9 (3.8)	8.7 (2.6)	<0.05	<0.01	0.2
Risperidone (n = 60)	23.8 (4.9)	17.9 (4.5)	11.7 (3.6)	9.1 (2.9)			
**PANSS negative score**							
Paliperidone (n = 60)	14.5 (5.6)	12.6 (3.7)	9.5 (3.1)	8.0 (1.7)	0.7	<0.01	1.0
Risperidone (n = 60)	14.5 (6.0)	12.8 (3.8)	9.6 (2.8)	8.2 (2.3)			
**PANSS general psychopathology score**							
Paliperidone (n = 60)	35.8 (6.4)	29.2 (5.3)	23.9 (4.5)	20.9 (3.6)	<0.05	<0.01	0.8
Risperidone (n = 60)	36.0 (6.5)	31.0 (5.1)	25.0 (4.0)	21.9 (3.0)			
**CGI-S**							
Paliperidone (n = 60)	5.4 (0.8)	4.6 (0.7)	3.3 (0.9)	2.5 (0.7)	0.1	<0.01	0.9
Risperidone (n = 60)	5.5 (0.8)	4.8 (0.8)	3.4 (0.8)	2.6 (0.7)			
**METH craving score**							
Paliperidone (n = 60)	7.5 (1.1)	6.5 (0.9)	5.6 (0.8)	5.2 (0.7)	<0.05	<0.01	0.1
Risperidone (n = 60)	6.5 (1.7)	5.9 (1.4)	5.3 (1.4)	5.2 (1.3)			
**BARS score**							
Paliperidone (n = 60)	0.6 (0.7)	0.5 (1.0)	0.8 (1.3)	0.6 (0.8)	0.9	<0.05	0.2
Risperidone (n = 60)	0.5 (0.8)	0.4 (0.6)	0.8 (0.9)	0.9 (1.2)			
**SAS score**							
Paliperidone (n=60)	0.1 (0.4)	0.8 (1.3)	1.8 (2.0)	1.8 (1.8)	<0.01	<0.01	<0.01
Risperidone (n=60)	0.1 (0.5)	1.4 (0.3)	3.1 (1.6)	3.1 (1.4)			
**Weight**							
Paliperidone (n = 60)	66.0 (12.6)	67.2 (12.5)	70.4 (12.0)	73.9 (12.0)	0.3	<0.01	1.0
Risperidone (n = 60)	64.5 (9.6)	66.0 (9.6)	70.0 (9.6)	73.2 (10.1)			

### Safety Data

**Figure 3:**
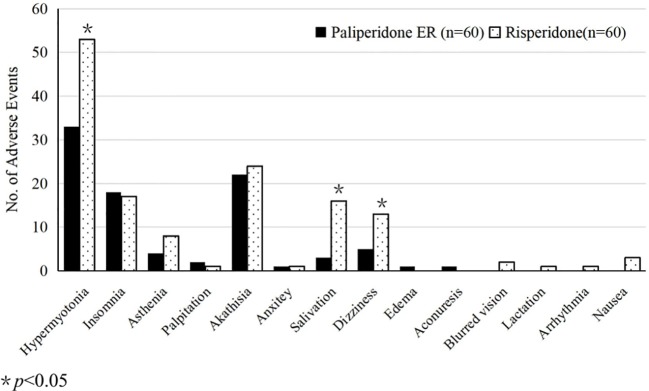
Clinical adverse events reported by study participants treated with paliperidone and risperidone.

The EPS (BARS and SAS scores) increased from baseline during treatment in both groups (*p* < 0.01); there were no statistically significant between-group differences in the BARS scores (*p* = 0.2), but there were statistically significant differences between the treatment groups in the SAS scores (*p* < 0.01; see **Table 2** for detailed results). Statistically significant differences were also observed between the two groups in the incidence of the following adverse events: hypermyotonia (33/60 or 55% of the paliperidone participants and 53/60 or 88.3% of the risperidone participants, *p* < 0.05); salivation (3/60 or 5.0% of the paliperidone participants and 16/60 or 26.7% of the risperidone participants, *p* < 0.05); and dizziness (5/60 or 8.3% of the paliperidone ER participants and 13/60 or 21.7% of the risperidone participants, *p* < 0.05). More patients in the risperidone group than in the paliperidone group reported asthenia (4/60 or 6.7% vs. 8/60 or 13.3%), but these differences did not reach the level of statistical significance (*p* = 0.2) (**Figure 3**). Body weight showed a statistically significant increase from baseline over time for patients in both groups (*p* < 0.01 for both), and the differences between the treatment groups were not statistically significant (all *p* > 0.05 for the medication and medication-by-time interaction effects; see **Table 2** for detailed results).

Changes in the patients’ blood chemistry and ECG were evaluated for the study completers (n=50 in the paliperidone ER group and n=51 in the risperidone ER group). These results did not change from baseline to day 25 in either group (all *p* > 0.05) except for serum prolactin, which increased from baseline to day 25 in both groups, but the increase was significantly greater in the risperidone group than in the paliperidone ER group (15.5 (25.8) vs. 29.7 (32.5) ng/ml, *p* < 0.05).

## Discussion

In the current study we found statistically significant reductions in clinical symptomatology, but including both positive and negative psychotic symptoms based on the PANSS total scores and on CGI-S scores, during treatment in both the paliperidone ER and risperidone groups. By the end of the trial, there were also substantial reductions in METH craving scores in both groups, with no statistically significant between-group differences in the reduced psychotic symptoms and METH craving scores.

The adverse event profiles of the two studied medications showed significantly lower rates of hypermyotonia, salivation, and dizziness in the paliperidone ER group than in the risperidone group. In the current study, prolactin levels also increased significantly, but the increase was lower in the paliperidone ER group than in the risperidone group. DA inversely regulates prolactin secretion from the pituitary gland; therefore, the prolonged blockade of potent D2 receptors on lactotroph cell membranes can increase prolactin levels. Antipsychotic drugs have different binding affinities for DA receptors on pituitary lactotrophs, and drugs with weaker binding affinities for DA receptors may result in lower increases in prolactin levels than drugs with stronger binding affinities (30). This may explain the weaker influence of paliperidone ER on prolactin levels (18). Paliperidone is also a weaker affinity for α1 and α2 adrenergic receptors, which may contribute to fewer adverse events than observed with risperidone (18, 20).

Individuals with MAP are out of touch with society, and ultimately, their social functioning is completely eliminated. Therefore, they would benefit from an early intervention approach for psychosis to reduce the risk of recurrent psychotic episodes or the development of a chronic psychotic disorder (31). We performed another clinical trial and found that paliperidone ER administration resulted in a reduced rate of psychotic symptom relapse among adults 12 weeks after acute METH detoxification treatment (32). Therefore, paliperidone ER had good efficacy and safety for both MAP treatment and relapse prevention.

There are a number of limitations in our study design that need to be discussed. The DSM-IV criteria for psychotic disorders were utilized to discriminate between substance-induced and primary psychosis in our study; however, differentiating between them is still difficult. A proportion of people who seemingly develop MAP have symptoms that persist for a month or longer (33) or that recur in the absence of recent METH use. It is unclear whether these cases of persistent MAP reflect a prolonged psychotic reaction or the precipitation of a primary psychotic disorder in vulnerable individuals. Therefore, we did not ensure that all the participants specifically had MAP. The study treatments were provided in a clinical ward, and the dosage and dose increases of both antipsychotic drugs were not fixed. Instead, the dose of the antipsychotic medication was modified (increased) until psychotic symptoms dissipated, and a broad range of ancillary medications were allowed. Thus, the effective dose of the two medications could not be compared. In addition, the study was not fully blinded for the doctors and patients but was for the investigators. Moreover, the study lacked a placebo control group; therefore, changes over time in clinical symptomatology could not be reliably distinguished from possible self-abatement of symptoms resulting from METH abstinence. There were only 15 women in the sample, so the findings may not be generalizable to women. Last, we did not obtain the number of patients deemed necessary by the power analysis, which is another potential source of Type II error and could lead to the suspicion that the drugs are in fact not equally effective.

## Conclusions

In summary, our study showed that paliperidone ER and risperidone have similar efficacy in the treatment of MAP; however, paliperidone ER had a more favorable adverse event profile than risperidone. In particular, extrapyramidal and prolactin side effects were less frequent or less intense. Therefore, paliperidone ER may be safer and better tolerated by patients when used as a treatment for MAP. The inclusion of a placebo control group or a placebo lead-in period and larger sample sizes in future studies may enhance our understanding of the observed effects and provide additional data on the efficacy, tolerability, and safety of these treatments.

## Data Availability Statement

All datasets generated for this study are included in the article.

## Ethics Statement

The study was reviewed and approved by the institutional review board of the Second Xiangya Hospital, Central South University, and written informed consent was obtained from the patients or, for patients with severe active psychosis, from their legally authorized representatives.

## Author Contributions

GW and XL: writing of article, acquisition of data, critical review of article, and analysis/interpretation. QD: contributed to the acquisition of data, analysis/interpretation, and final approval for publication. MC: writing of article and critical review of article. WH: contributed to the conception and design, acquisition of data, analysis/interpretation, and final approval for publication. XL: contributed to the conception and design and writing of article. FD: writing of article, acquisition of data, and analysis/interpretation. XO: contributed to the analysis/interpretation, and final approval for publication.

## Funding

The open access fee was paid by the authors. This study was supported by grants from the Wuhan Health and Family Planning Commission (WG16A02, Bao-Liang Zhong, PI; WX17A07, XL, PI), Wuhan Medical Research Program (WX19Z31, WG, PI), Hubei Natural Science Foundation (2018CFB334, KZ, PI) and Ministry of Public Security, the Key Program of the National Natural Science of China (81130020, WH, PI), and the National 973 Program (2015CB553500, WH, PI).

## Conflict of Interest

The authors declare that the research was conducted in the absence of any commercial or financial relationships that could be construed as a potential conflict of interest.
